# On the Use of Water in Surgery

**Published:** 1851-07

**Authors:** Alphonse Auguste Amussat

**Affiliations:** Paris


*To be Continued.*

---

ART. II.— ON THE USE OF WATER IN SURGERY. *By Alphonse Auguste Amussat, of Paris ; published at Paris, Jan.* 1851, *and translated from the French, by Frank H. Hamilton, Prof. of Surgery in the University of Buffalo.*

(*Continued from page* 773.)

6. *What are the inconveniences which have been attributed to this means?*

If we refer to the cases which have been reported, we shall find that the first impression of cold water upon an injured part, especially if there is a wound, is generally very painful, and the more so in proportion as the temperature is lower and the condition of nervous surexcitation more distinct. Sanson says he has seen many persons wounded by firearms, in whom cold water produced such intolerable pains as compelled him to suspend its use. Guthrie laid it down as a rule, that if cold water was disagreeable to the patient it must be renounced. M. Patry has noticed in the cases which he have been able to verify in the hospitals of Paris and in civil practice. Gradually, however, the patient becomes accustomed to the cold water, so that it ceases to be painful, and remains thus until the inflammation subsides. Then the injured parts by returning to their normal state recover their natural sensibility, and the patient complains of pain: this is ordinarily the sign upon which we depend to suspend the water and to commence the cerate dressings. Such are the effects of cold water, while on the contrary, water at 18° or 25° (64° or 77°F.) does not occasion any painful sensation even at the first, yet it calms the excitement generally as well as the cold.

The application of cold water, especially in winter, produces horripilations and shiverings which continue more or less time. I have lately seen in one of the hospitals of Paris, a man who had had his hands crushed in the cog wheels of machinery. The surgeon, after having amputated two fingers, and approximated the flaps with diachylon plasters, submitted the hand to continued irrigations of cold water. The patient assured me that during the eight first days he experienced constant chills, despite all the means employed to keep him warm; but from this time he was able to endure the cold water without inconvenience for more than a month longer. When I saw it, on the sixth week, ordinary dressings had been commenced, and it was nearly cured. Such cases are common.

Sanson says he saw a female with whom a superficial burn seemed to indicate cool applications, and who was seized with tetanus soon after their use had been commenced. Again, M. Patry has reported the case of a young girl whom he subjected to applications of cold water upon the head, for a complicated fracture of the frontal bone; the mother having discontinued the applications on the fourth night, tetanus immediately supervened, and did not cease until the water was re-applied as at first. Thus we see that this grave complication may be produced both by the application of cold water and its sudden suspension.

"Mortification of the tissues, the accident which seems most to be feared from refrigeration so long continued," says A. Berard, "is extremely rare, and we have not seen it even in parts submitted to running cold water, unless they were already disorganized by the violence of the contusion. It is more easily induced in the extremities of the limbs, and I have had the misfortune of seeing it twice occur upon the great toe.

"I will not say whether in this case the gangrene was produced by the contusion itself, or by the refrigeration; certainly I have seen wounds of the same nature, treated by the ordinary means, followed with gangrene of one or several phalanges, while others, submitted to irrigation, have been exempt.

However, I believe I shall be able to establish the following principles. Whenever the contusion, complicated with a wound, leaves sufficient soft parts intact to permit the circulation to go on with ease through the whole extent of the limb, gangrene is not to be feared; but if in the thickness of the limb only a little structure remains which the contusion has not disorganized, even although the extremity beyond is perfectly uninjured, gangrene is not the less to be apprehended in the extreme portions. Cold water then acts without doubt with too much energy, and under its sedative influence the circulation is retarded, and perhaps even completely suspended in the tissues which the contusion has spared, and life is extinguished in every part which has lost its vascular connection with the rest of the body."

Goursaud reports a case of Guyenot's, in which ice having been applied an hour or two upon a strangulated crural hernia, the hernia was not reduced, and the surgeon, obliged to resort to an operation, found the epiploon frozen; the intestinal knuckle was, however, not injured, and the patient recovered.

I have notes of the case of a patient affected with a phlegmonous erysipelas of the arm and forearm, with whom the continued application of ice produced a solidification of the pus, so that for its removal it became necessary to resort to shampooing, and very firm graduated compression.

My father has been often consulted on account of a gangrene which he has thought ought to be attributed to the employment of very cold water; among the cases of gangrene which I have myself seen, there are several which must be ascribed to the same cause.

I have collected also several cases of patients who having been submitted to irrigations with cold water, have suddenly died with some nervous malady. What part does the cold play in the development of these phenomena? Without being assured that it is the principal cause, I believe I can at least say that it has some agency.

"Who will affirm," says M. Richet, "that the application of a powerful refrigerant upon a large surface will not, by repelling inward upon the viscera the blood which originally abounded in the diseased part, occasion congestions, and give birth to those complications to which I have alluded! The facts are every where to be seen, and the practitioner ought to profit by them."

"It is well known," says Sanson, "that cold applications may cease to be useful, and may even become hurtful, by rendering the flesh œdematous, and pale, and causing it to become irritable when suppuration is established in the wounds. Sometimes also they entirely prevent the development of inflammation to such a degree as that at the end of twelve or fifteen days the wound is still in nearly the same condition as at the moment of the accident."

M. Apvrille reports a fact upon this point, which occurred in the service of M. Jobert. A woman had received a blow from the horn of a cow, which had torn extensively the skin and superficial muscles of the abdomen; cold water compresses were applied and renewed every ten minutes; when this mode of treatment had been pursued for some time, the wound was found to have made no progress toward a cure, and the cold was suspended. The next day a violent inflammation ensued; again the cold water compresses were applied, and the wound returned to its original condition. A renewed suppression of the compresses was followed by a yet more intense inflammation. Gradually a flabbiness supervened, and the patient died.

M. Cloquet has remarked to me that he has observed the phenomena noticed by Sanson, in debilitated subjects, when cold has been used perseveringly.

"Cold," says Tanchou, "is only suitable for the young and robust; with feeble persons, the very old, and with infants, it is always injurious." This proposition is the more true as the time of the application is more prolonged.

One will ask, perhaps, why the accidents of which we speak are not observed more frequently? I answer, that in general surgeons have not taken care to note them, and farther, the temperature and the quantity of water employed in a given time being seldom indicated in the report, it is difficult to understand exactly the degree of refrigeration produced, and whether, therefore, the accident ought or ought not to be ascribed to the cold.

In fact, as to the direct effect of the cold in these cases, M. Nivet thinks that water a little below the temperature of the part, acts not so much by abstracting caloric, as by diminishing the vascular circulation.

If we examine now the condition in which patients are placed, we shall not fail to be convinced that cold, which is easily borne by a man strong, robust, and healthy, having proper exercise, and taking sufficient nourishment, cannot be so well borne by a man suffering, obliged to keep his bed, breathing an air more or less impure, and not using his ordinary but an insufficient nourishment.

We see from what precedes, that if cold water possesses some great advantages, it has also many inconveniences, and under certain circumstances it becomes even dangerous. We ought then to prefer tepid water, which calms the pain, and produces the desired effect of subtracting the caloric without exceeding the proper limits, and without exposure to any of the inconveniences of cold water, such as chills, too sudden suppression of the inflammation, and especially gangrene.

I must not omit to say, in closing my reflections upon the use of water, that there are certain constitutions in which it does not present favorable results, whatever precaution we may take. In those cases, which it is nevertheless impossible to foresee, we ought, when the idiosyncracy becomes known, immediately to abandon it, and substitute for the water, perhaps the *appareil a incubation* of M. Guyot, perhaps the *appareil ouate* of Dr. Burggraeve, of Ghent, or other such means as now may be regarded as suitable.

The diseases in which water has been employed with advantage, and which constitute the subject of the numerous cases in my possession, are *simple inflammations, erysipelas, burns, ulcers, gangrene, wounds simple and contused, gunshot wounds, wounds from operations, amputations, &c., hemorrhages, contusions, affections of joints, hernias, diseases of the eyes, diseases of the genital and urinary organs of males and females.*

The application of water upon any portion of the body does not dispense with the necessity of ordinary medical means. When indicated, we are still to resort to bleeding, purgatives, emetics, &c. Water, acting locally, calms, it is true, the inflammatory symptoms, in the part subjected to its action; but it cannot neutralize the effect already produced upon the whole system. Nevertheless, when employed as soon as the lesion is produced, it is found in a great many cases sufficient in itself alone to prevent all reaction, and may constitute the sole treatment. It is easily comprehended that the case is altered when several hours or perhaps several days have elapsed since the occurrence of the accident; the general symptoms which have become developed may then demand the employment of those medical means of which I have just spoken.

In this part of my essay, I have only spoken of simple water, which will suffice in a great number of cases. Nevertheless, I think that we may obtain some advantages from water charged with medicinal substances; but this question is one of those which I am obliged to pass over at present, and reserve for a future time.

### CHAP. III.

### PRINCIPAL MODES OF APPLICATION OF WATER IN SURGERY.

The various modes in which water has been used in surgery, may be arranged under three grand divisions, viz., *water-dressing, irrigation, immersion.*

*Water-Dressing.*—In surgery we give the name "dressing" to objects which are applied to a wound, and the name "water-dressing" to these same objects wetted and kept moist during more or less time.

Without intending here to enter into a history of "water-dressings," we will nevertheless say that this was the mode of using water adopted by Lombard, Percy, Larrey, Treille, &c., and to which we have already referred.

The ancients, who preferred usually a gentle moistening, with a mild temperature, employed, in place of lint, discs of sponge, of various thicknesses, which they laid carefully upon the wound, so as not to incommode the part by its weight. The application of the sponge was either mediate or immediate, and as it absorbed much water it retained its moisture a long time. They took care also to supply the water which it lost by absorption or evaporation by renewing it from time to time. By these means they were able in many cases to maintain about the injured part such a suitable moisture as contributed both to the comfort of the patient, and to the cure.

Percy, who has studied with care the effects of water in a great number of affections, and who has noticed the inconveniences inseparable from cataplasms and fomentations, has made the following experiments with a view to find a material which would advantageously replace them, and at the same time retain the moisture and the warmth: "If we take," says he, "a piece of linen, another of common cotton cloth, a third of fustian, a fourth of English dressed flannel, and a fifth of soft flannel, all of the same size, and saturate them with distilled water; if now we suspend them side by side, at the same height, exposed to the same degree of heat on a summer's day, we shall find that the linen will dry almost immediately, the cotton soon after, the English flannel will be three times longer, and the soft flannel will remain wet some hours after the others are dry.

It is true that the soft flannel will have absorbed the most, and progressively each will be found to have absorbed less and less until the linen will be found to have absorbed the least of all: but by multiplying the pieces, or by folding them together, and thus making them absorb the same quantity of water as a simple piece of soft flannel, we shall still see that the linen with all its folds will dry six times as soon as this flannel. It will be proportinually the same also, with the other fabrics mentioned.

Without doubt the best vehicle for water used as a topical application, and that which ought to supply the place of cataplasms, fomentations, &c., is soft flannel, which, in addition to its property of retaining much more of this liquid than any other tissue or material, prevents also its becoming suddenly cooled, being, as we say, a very bad conductor of caloric."

While we recognize the advantages which soft woolen flannel (*molleton*

*de laine*) possesses over other fabrics, we by no means consider it free from every objection, especially in winter, or in cold and damp countries; Percy himself has observed this, and when he wishes to relax and soften a part and retain a perpetual moisture, (in a word to obtain all the advantages of cataplasms without their inconveniences,) he advises to saturate the linen with tepid water, and then lay over it a piece of oiled bladder, glazed linen or taffeta, and even parchment softened in sweet oil.

The difficulty of maintaining water at a mild and agreeable temperature in cold and humid countries has without doubt led Percy to cover the pieces holding the water, with an impermeable tissue; the same consideration has probably determined Liston, and, after him, the surgeons of the hospital of the University of London, to give the preference to this kind of water-dressings, which they employ almost exclusively.

M. Morton informs us that in this establishment they have banished every form of greasy dressings, and that they proceed as follows, in employing simple water or occasionally water medicated: they take a thick piece of charpie, (lint,) which is to be moistened more or less with tepid water, according to the indications; they place it on the wound, and cover it with oil cloth to prevent the too rapid evaporation of the liquid. From time to time, usually every three or four hours, the lint is removed and dipped in water, and if the suppuration is very abundant, or of a bad quality, it is renewed each time.

Josse and A. Berard employed at first compresses wet with water; but they soon became dissatisfied with this method. The part under treatment was found to be subject to frequent alternations of heat and cold, since the moistened compress became rapidly heated when not regularly renewed; A. Berard also remarks that such constant attention as this mode demands is sufficiently difficult during the day, but during the night it is almost impossible. It is easily comprehended how many circumstances may interpose to prevent a regularity in the temperature, and to induce a transition from cold to heat or from heat to cold. Hence these two surgeons were led to the adoption of continued irrigations, and this was a great step in the progress of surgical therapeutics.

M. Malgaigne gives the name of *intermittent irrigation* to lint moistened and applied as above.

But water-dressings, that they may prove useful, and be exempt from the inconveniences heretofore urged against them, must fulfill the following indications.

1. They must permit the pus to escape freely as fast as it is formed, and to be absorbed by the dressings.

2. They must be kept constantly moist.

3. Evaporation must be prevented, lest the part should become chilled, or in other words a uniform temperature must be preserved.

We think we have accomplished these several indications by a water dressing which my father and myself have much used, formed of four pieces of different tissues regularly superposed, which pieces may be called respectively the *sifter*, (*crible*,) the *absorbent*, the *humectant*, and the *inevaporant*.

*Sifter.*—The *sifter* is a tissue perforated with a great number of holes, to allow the pus to escape as fast as it is formed, and to separate the wound from the substance which absorbs the purulent matter.

The necessity of not permitting the pus to rest upon the surface of the wound has long been understood, and surgeons have been accustomed to cover the wound with a perforated compress spread with cerate. But the perforated linen used in hospitals and in civil practice does not fulfill the indications which we seek to obtain. Indeed, the holes being too small and too wide apart, and the pus becoming mixed with the cerate, the perforations are easily closed, and it serves no purpose whatever. With a view to remedy this inconvenience, my father first laid aside the cerate, and dipped the compress in water, but the pus still accumulated as before; he then used ordinary gauze instead of wetted compresses. The spaces which separated the threads in this tissue were too small, and the difficulty was not remedied.

Finally he tried common tulle with very open tissue, which perfectly answered the indication. This is the tissue which he decidedly prefers, and which he has named the "sifter."

If tulle cannot be obtained, we may use linen with larger and more frequent perforations, or strips of linen arranged in quadrille form, with larger spaces than are usually left.

*Absorbent*—Isolation of the wound being obtained, in the manner which we have described, my father next sought what would be the best means of absorbing the pus as fast as it was formed.

After having tried many tissues, he has concluded that old rags from linen or cotton, sufficiently worn, answered this purpose best. He places a disk of proper size, after having moistened it with tepid water, over the *sifter*. This piece of dressing has received the name of *absorbent*.

*Humectation.*—Humectation being the principal indication to answer in water-dressings, it became important to ascertain what substance would best accomplish this.

After having tried, successively, discs of sponge, of soft flannel, of lint, of soft cotton, of agaric, &c., my father has finally given the preference to punk,

(*amadon,*) prepared without saltpetre or gunpowder. This substance, with little volume, absorbs much water, is soft to the touch, and yields its water to the *sifter* more easily than those which we have enumerated; We therefore generally prefer it. This piece of dressing has received the name of *humectant.*

*Inevaporant, or impermeable tissue.*—A last condition remains to be attained, that is, to prevent the evaporation of the liquid as much as possible. This indication has already been observed and answered by Percy, who placed over the compresses wet with water an impermeable tissue. In In the same manner also we protect the water-dressings. Almost any impermeable tissue will answer the purpose, but It is proper to mention that as in the country the means are not always so abundant, a hog's bladder soaked in oil does very well. If we desire to prevent the evaporation completely, we must not neglect to make this cover larger than the other portions of the dressing, otherwise there will be along the borders of the dressing a slow evaporation, producing a coldness, especially in winter.

Dressings of the fingers, of the great toes, and of the penis, ought to be completely enveloped in the impermeable tissue so as to form a sac within which the moisture shall be retained. A piece of intestine or of caoutchouc made into a sac and placed over the member, will serve an excellent purpose.

An emollient effect being that which we wish generally to produce; we ordinarily use in these cases soft and tepid water at a temperature of 18° or 25° C., (64° or 77° F.) The liquid acquires soon the temperature of the part, especially if the impermeable tissue completely envelops the dressings, but if it does not, evaporation occurs, and a sense of chilliness results.

As to the time during which the application is to be continued without renewal, this must vary according to the effects which we wish to obtain, and according to the state of the parts. If the inflammation is active the dressings should be often renewed, and so also if the production of pus is very abundant. In simple cases, however, it will be sufficient to change the dressing every four or six hours.

This mode of dressing, less powerful than irrigations and immersions, has the advantage of permitting the patient to attend to his occupations, when there is nothing to contra-indicate it.

When we wish to discontinue the water-dressing, it must not be done suddenly, but gradually, so as not to incur the hazard of a return of accidents. We must begin by diminishing the quantity of water employed in moistening the dressings, and then little by little make it cease altogether.

Compared with other modes of dressing, the water-dressing is infinitely superior, even to cataplasms. We observe that in the great majority of cases it possesses all their advantages without their inconveniences, and as it can be easily maintained without being disturbed, it saves much pain to the patients.

I have already seen the dressing which I have now described used in a sufficient number of cases to induce me to hope that it will soon be preferred to fomentations, to compresses of lint and other tissues soaked in water, &c. I do not, however, reject, absolutely, cataplasms, which may be very useful in many cases, if properly employed, that is to say, they ought to be very moist and frequently renewed, especially when there is a pretty abundant suppuration.

